# Acute and chronic effects of gold nanoparticles on sperm parameters and chromatin structure in Mice

**Published:** 2016-10

**Authors:** Mahsa Nazar, Ali Reza Talebi, Mohammad Hosseini Sharifabad, Abolghasem Abbasi, Arezoo Khoradmehr, Amir Hossein Danafar

**Affiliations:** 1 *International Campus, Shahid Sadoughi University of Medical Sciences, Yazd, Iran.*; 2 *Research and Clinical Center for Infertility, Shahid Sadoughi University of Medical Sciences, Yazd, Iran.*; 3 *Department of Biology and Anatomical Sciences, Shahid Sadoughi University of Medical Sciences, Yazd, Iran.*

**Keywords:** *Nanoparticles*, *Sperm*, *Chromatin*, *Mouse*

## Abstract

**Background:**

The particles in the range of 1-100 nm are called nanoparticles. Gold nanoparticle is one of the most important metal nanoparticles with wide usage.

**Objective:**

This study investigated the effects of gold nanoparticles on sperm parameters and chromatin structure in mice.

**Materials and Methods:**

In this experimental study, 72 male bulb-c mice were divided into 9 groups including: 4 Sham groups (Sc 1-4), 4 experimental groups (Au 1-4), and 1 control group (C). Experimental groups received 40 and 200 µg/kg/day soluble gold (Au) nano-particles for 7 and 35 days, by intra peritoneal injection, respectively. Sham groups were treated with 1.2 mM sodium citrate solution with 40 and 200 µg/kg/day doses for same days and control group did not receive any materials. Motility and Morphology of spermatozoa were analyzed. Chromatin quality was also evaluated using AB (Aniline blue), TB (Toluidine blue) and CMA3 (Chromomycin A3) staining methods.

**Results:**

The sperm analysis results showed that motility and morphology of sperm in experimental groups (especially in groups that have been treated for 35 days with nano-particles) had significant decrease in comparison with control group. TB, AB and CMA3 results showed a significant increase in abnormal spermatozoa from all Au-treated groups.

**Conclusion:**

Gold nano-particles firstly can reduce the sperm parameters such as motility and normal morphology and secondly affect sperm chromatin remodeling and cause the increase instability of chromatin and also increase the rate of sperm DNA damage. These deleterious effects were more obvious in maximum dose and chronic phase.

## Introduction

Nano is a Greek prefix meaning 10^-9^ and nowadays this word is used in Nanotechnology (1). Nanotechnology as an emerging technology has been expected to influence the areas of Information technology, medical treatment and environment protection (2). Unique characteristic of nanoparticles in comparison with their raw material is the fact that their diameters are less than 100 nm and cause high ratio of surface to volume (3). Gold is an expensive metal which its major application is on jewelry. On the other hand, the researchers have found the medical applications of gold salts like in dentistry and treatment of rheumatoid arthritis (4-5). 

The nanoparticles are capable of penetrating inside the cells and combining with DNA molecules due to their extremely small sizes and unique physical and chemical properties which are different from those of larger materials made up of the same components. Furthermore, the germ cells may interfere with fertilization process and formation of fetus (6). In some experimental studies, the ability of gold nanoparticles with different sizes in penetration into the DNA molecule and positioning in DNA large groove has been shown (7). 

It is shown that these nanoparticles have spermatotoxic effects. They suppress the movement of sperm cells and prevent the decondensation of sperm chromatin (8). It has been also shown that low levels of chromosome mutations induced by gold nanoparticles in initial spermatocytes cells do not result from direct impact of these nanoparticles on genetic structure of masculine gems cells, but rather from inducing their impacts by creating disorders in function of the enzymes which are in charge of repairing spontaneous chromosome damages (9). During transition of spermatozoa, they undergo vast changes which prepare them for fertilization (10). However, sperm chromatin and DNA are very sensitive to exogenous and endogenous stresses during the above mentioned journey.

Unlike somatic cell chromatin that contains histones, the spermatozoa have an specific protein called protamine. During spermatogenesis, about 85% of histones in human and 95% in mouse are replaced by protamines and the reason of remaining of the rest histones is probably because of the role of this genes in fertilization, early embryo development and expression of a number of genes, including influencing genes (11). 

Studies have indicated that in the cases of fertilization of ovum by a DNA-damaged sperm, the rates of implantation and pregnancy are significantly reduced (12-14). Although, damaged paternal DNA can be repaired during growth of embryo, but in cases of large amounts of sperm DNA damages, the repair is not enough and the process of reproduction will be affected and finally these damages may cause after birth defects (15). Causes of sperm DNA damage such as causes of male infertility are complex and related to intrinsic and extrinsic factors (12, 16-18). Zakhidov reported that gold nano-particles cause abnormal chromatin condensation in mouse sperm (6). According to another study selenium nanoparticles can impact the sperm DNA integrity (19). 

In spite of widespread application of nanoparticles, there are insufficient information on their impact on human health and environment. Hence, evaluating the probable adverse side effects of gold nanoparticles and their impacts on various sperm parameters as well as chromatin and DNA integrity in men who are exposed to gold nanoparticles are very important. So, the purpose of present study was to investigate the acute and chronic effects of gold nanoparticles with different doses on sperm parameters and chromatin structure of mouse as an experimental model.

## Materials and methods

**Nanoparticles**

Spherical shape gold nano-particles with a diameter of 10-30 nm were purchased from Tehran University in a form of prepared Colloidal solution with black color and concentration of 1000 ppm per 100 ml for injection to the mice.

**Animals and experimental design**

In this experimental study which was done in research and clinical center for infertility, Yazd, Iran during 2015-2016, totally 72 male bulb-c mice with age of 5 wks and mean weight 22±4 gr were randomly divided into 9 groups (n=8 in each group) including 4 sham, 4 experimental and 1 control groups (C). The sham animals treated with sodium citrate solution of 1.2 mM in doses of 40 and 200 μg/kg/day for 7 and 35 days (Sc 1-4 respectively) (20). The treated groups received gold nanoparticles solution with the same doses and same times (Au 1-4 respectively) by i.p injection and the control group did not receive any material during experiments (20). The mice were kept for at least 2 wks before experiments at standard optical (12 hr light/dark) and appropriate thermal condition (22-25^o^C) in the clean cages and feed by special food and access to water ad libitum. 

**Epididymal sperm sampling**

One day after the last injection in each group, the mice were sacrificed and the cauda epididymis of each animal was removed and placed in a petri dish containing 1000 µl of Ham's F10 medium. The dishes were incubated for 30 min at 37^o^C and 5% CO^2^ (21). Sperm motility and morphology were analyzed and chromatin quality was assessed using aniline blue (AB), toluidine blue (TB) and chromomycin A3 (CMA3) staining methods.

**Sperm analysis**

For sperm motility we used the standard method of Makler chamber (Sefi Medical Co., Haifa) and evaluated by optical microscope (Olympus, Tokyo, Japan) at 20× magnification. The spermatozoa were divided into immotile (grade d), progressive motile (grades a+b) and non-progressive sperm (grade c). To investigate sperm morphology, we used Papanicolaou staining. Briefly, the sperm smears were fixed in ethanol-ether solution (1:1) for 4 min and then the slides were stained according to the WHO guideline (22).

**Assessment of chromatin quality**

**Aniline blue staining**

Histone protein has many lysine residues which react with acidic stains like AB and become blue in color. Spermatozoa with residual histones are considered as AB^+^ or immature cells. To do this test, after air-drying of smears, the fixation was done by 3% of glutaraldehyde in phosphate buffer for 30 min. In the next step, samples were stained by 5% solution of AB (Merck, Germany) in acetic acid 4% with the pH of 3.5 for 5 min. After washing by distilled water and mounting by DPX (Merck, Germany), the slides were examined by light microscope (Olympus, Tokyo, Japan) at 100× magnification. The percentage of colorless sperm cells (mature) and blue sperm cells (immature) were obtained (23).

**Toluidine blue staining**

TB is a metachromatic dye binds to the exposed phosphate groups of DNA and shows both chromatin condensation and DNA integrity (21). To do this test, the smears were air-dried and then fixed by ethanol-acetone (1:1) at 4^o^C for 30 min. For each sample, acidic hydrolysis was done by HCl solution (0.1 N) at 4^o^C for 5 min, and then washed by distilled water 3 times for 2 min. The staining was done by 0.05% TB in 50% Mcilvian buffer for 10 min and then evaluated by light microscopy at 100× magnification. In this staining we have a range of colors: light blue (sperm with normal chromatins), dark blue (sperm with slightly abnormal chromatin) and purple (sperm with sever chromatin abnormality) (23).

**Chromomycin A3 staining**

CMA3 is used for the evaluation of degree of protamination in spermatozoa (24). After drying of smears, we fixed slides by Carnoy's solution (methanol and Glacial acetic acid, 1: 3) for 10 min at 4^o^C and then we stained them with (0.25 mg/ ml) in McIlvain buffer (Sigma, USA) for 20 min. After washing and mounting of slides, spermatozoa were counted under florescent microscopy (BX51, Olympus, Tokyo, Japan) with a 475-nm filter and X100 eyepiece magnification and the results were expressed as percentage of CMA3+ spermatozoa (24).

**Statistical analysis**

After collecting data, we used the Spss software version 18. Differences between variables with normal distribution were analyzed by ANOVA test and between each 2 groups were determined by post hoc tests. The term ‘statistically significant’ was used for P-value ≤ 0.05.

## Results

**Sperm parameters**

The progressive motility and non-progressive motility showed significant differences between all groups (p=0.000 and p=0.002 respectively). The treatment of mice with gold nanoparticles causes a significant increase in abnormal sperm morphology (p=0.000). In microscopic assessments, the different forms of sperm morphological defects such as deformed heads, bending and fracture of the necks and coiled tails especially in groups treated with 40 and 200 μg/kg/day of gold nanoparticles were observed. It should be noted that the duration and dose of injection have effect on sperm parameters. In other words these effects were more obvious in maximum dose (200 µg/kg/day) and chronic phase (35 days). The results of sperm parameters analysis of 9 groups are listed in table Ι.

**Chromatin quality**

**Figure 1:**
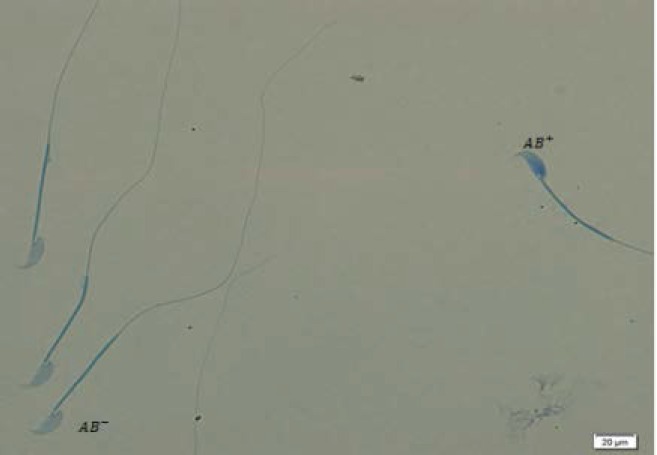
Aniline blue staining in 200Au/35 group. Normal spermatozoa are seen in light blue and abnormal spermatozoa are seen in dark blue ×100

**Figure 2:**
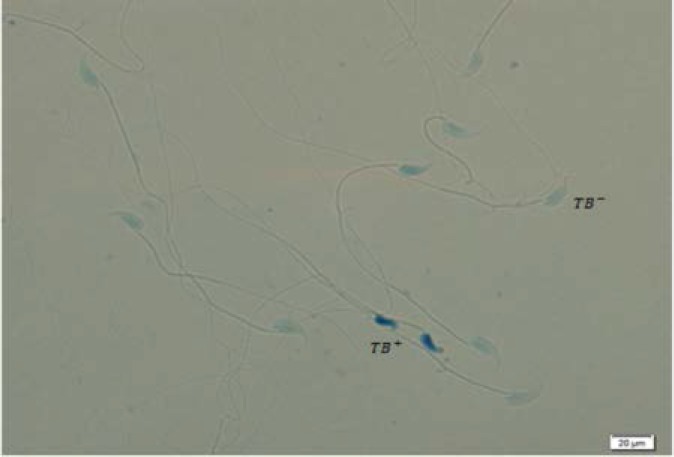
Toluidine blue staining in 200Au/35 group. TB^+^ spermatozoa are dark blue and TB^-^ spermatozoa are seen in light blue ×100.

**Figure 3:**
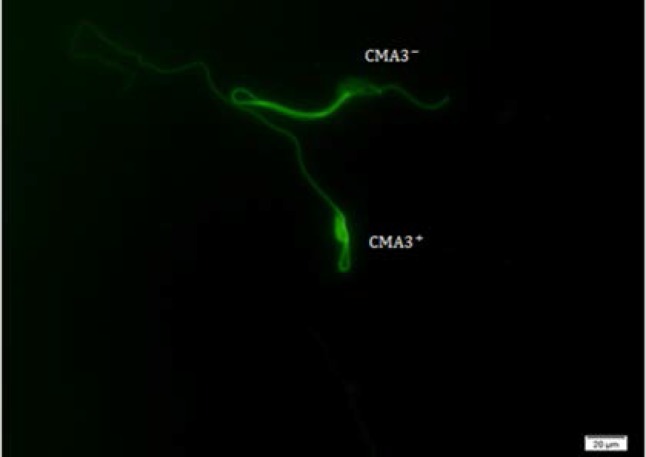
CMA3 staining in 200Au/35 group. Abnormal sperm CMA3^+ ^is seen in bright green and normal sperm CMA3^- ^is seen in non-bright green ×100

As shown in table ΙΙ, the rates of immature spermatozoa with abnormal chromatin (AB^+^) in 200 Au/7D and 200 Au/35D groups were significantly higher than sham and control groups with p<0.05 (Figure 1). The results of TB staining also showed a significant increase in abnormal spermatozoa in Au-treated animals (p=0.003). There were significant differences between 40 Au/7D group and control/Sc, 40, 35 (91.38±6.88, 98.00±1.41, 98.25±2.76, respectively; p<0.05) (Figure 2). In CMA3 test, the nuclei of spermatozoa with no brightness (CMA3^-^) and spermatozoa with bright nuclei (CMA3^+^) were counted by fluorescent microscope. The rates of CMA3^-^ sperm cells were difference between all experimental and sham/control groups. In this test the difference was significant between Au, 200, 35 with control, sham Sc, 40, 35 and sham Sc, 200, 35 (97.13±1.45, 99.75±0.46, 100.00±0.00, 99.63±0.51, respectively, p<0.05) (Table ΙΙ) (Figure 3). 

**Table Ι:** Mean and standard deviation of sperm motility and morphology in groups

	**Control (C)**	**40Sc/7D (sc 1)**	**200Sc/7D (Sc 2)**	**40Sc/35D (Sc 3)**	**200Sc/35D (Sc 4)**	**40Au/7D (Au 1)**	**200Au/7D (Au 2)**	**40Au/35D (Au 3)**	**200Au/35D (Au 4)**	**p-value**
Total motility	30.81±5.49	38.63±5.83	21.49±6.70	27.62±7.41	23.75±7.87	22.12±6.65	26.13±10.75	18.37±6.75	15.94±6.39	**0.000**
Normal morphology	65.63±5.63	64.88±7.68	69.00±4.34	51.25±9.42	57.63±8.36	45.50±9.59	50.88±8.54	25.63±13.00	29.63±6.63	**0.000**

P<0.05 represents a significant difference between groups. All data are present as mean ±SD.

D: treatment days

Sc: sham group

Au: experimental group

**Table ΙΙ:** Characteristics of sperm nuclear integrity in groups

	**Control ** **(C)**	**40Sc/7D ** **(sc 1)**	**200Sc/7D ** **(Sc 2)**	**40Sc/35D ** **(Sc 3)**	**200Sc/35D ** **(Sc 4)**	**40Au/7D ** **(Au 1)**	**200Au/7D ** **(Au 2)**	**40Au/35 ** **(Au 3)**	**200Au/35 ** **(Au 4)**	**p-value**
AB^-^	98.13±1.45	94.75±3.32	96.25±2.55	97.50±1.41	96.75±1.58	94.63±2.20	98.13±1.64	95.00±2.72	93.00±2.13	0.000
TB^-^	98.25±2.76	92.38±3.85	94.25±2.31	98.00±1.41	96.88±3.22	91.38±6.88	96.00±5.01	96.50±2.00	95.00±2.00	0.003
CMA3^-^	99.75±0.46	97.50±1.51	97.88±2.90	100.00±0.00	99.63±0.51	97.38±2.20	99.75±0.46	98.50±1.06	97.13±1.45	0.000

P<0.05 represents a significant difference between groups. All data are present as mean ±SD.

D: treatment days Sc: sham group Au: experimental group

## Discussion

According to our results, the gold nanoparticles firstly can reduce the sperm parameters such as motility and normal morphology and secondly impair sperm chromatin remodeling leading to increase instability of chromatin. It also increased the rate of sperm DNA damage. Regard to the sperm parameters, we showed that the percentage of sperm with progressive motility decreased especially at the dose of 200 μg/kg/day for 35 days. 

Because the duration of mouse spermatogenesis is about 31 days and to ensure that the nanoparticles effect on all of the cells in the process of spermatogenesis, we administrated them for 5 weeks. Furthermore, the impact of these nanoparticles on previously produced epididymal sperm cells was evaluated due to formation of disulfide bonds between adjacent protamines in chromatin structure of the sperm which takes place after one week in epididymis. It was observed that, epididymal spermatozoa are also influenced by gold nanoparticles.

The first part of our results showed that gold nanoparticles can affect different sperm morphological and motility parameters. In fact, treated groups showed more percentage of spermatozoa with abnormal morphology and low motility in comparison with sham and control groups. Taking together, the highest reduction in sperm motility and normal morphology was seen at dose of 200 g/kg/dayµ and duration of 35 days. It should be noted that the most abnormalities in sperm morphology were observed in tail (the twisted tail) and then in sperm head. Zakhidov *et al* demonstrated that the gold nano-particle shows spermatotoxic effects and may impact mouse spermatogenesis (6). 

In accordance to our results, Wiwanitkit *et al* in a study on donor sperm stated that the gold nano-particles show dose-dependent effects on sperm motility (8). Also, like our results, Moretti *et al* showed that both silver and gold nanoparticles affect the sperm motility in dose-dependent manner (28). Of course, it should be noted that the studies indicating the effects of gold nano-particles on male reproductive system are very limited and the majority of researches were done on other metal nano-particles like silver, zinc and selenium. Grodzka-Ostrowska *et al* also showed that although silver nano-particles can reduce the count of spermatozoa, but these particles do not decrease the sperm normal morphology (22). 

However, we saw different forms of sperm abnormalities such as head and tail malformations in gold-treated animals. Another study conducted by Rezvanfar *et al* showed that nano-selenium changes sperm parameters, including morphology, motility, viability and count (19). Also, in another study it was shown that zinc nanoparticles may affect mouse spermatogenesis and cause a significant decrease in sperm motility and increase in abnormal sperm morphology (1).

In recent years, gold nanoparticles have attracted particular attention and have found widespread use in scientific research, industry, medical, and especially to recognize and destroy cancer cells (25). Spermatogenesis is a complex process and is very sensitive that different factors can affect its quality (26). Nano-particles have negative effects on many organs, such as testes (24). Experimental in vivo studies have shown that nano-particles may easily transfer through the blood-brain and blood-testis barriers (28). 

Although, about gold nanoparticle, the exact mechanism of toxicity is not clear yet, but, it is demonstrated that this particle can incorporate to the major groove of DNA and cause different abnormalities in cells (29). In etiological terms, it should be mentioned that the gold nanoparticles can bind to DNA molecules and impact on chromosome repairing enzymes. In addition, the deleterious effects of other nanoparticles, such as increasing ROS production, production of mutagenic molecules may also come true about gold nanoparticles (30).

Consequently, more investigations are demanded in this area. In present study we used the cytochemical staining like AB, TB, and CMA3 to evaluate the quality of sperm chromatin. The results of AB, TB, and CMA3 tests showed a significant difference between treated and controls. Since AB indicates the presence of excessive histones, TB indicates the amount of chromatin condensation and CMA3 test indicates the protamine deficiency, it can be concluded that the process of spermiogenesis is affected by gold nanoparticles. In this part, the greatest impact was also observed in a dose of 200 g/kg/day and duration of 35 days. So, regard to the effects of nanoparticle on sperm chromatin, we can say that gold in the form of nanoparticle may cause increase residual histones, DNA fragmentation and protamine deficiency in mouse spermatozoa as an experimental model. 

In accordance to our results, Zakhidov *et al* investigated the effects of gold nanoparticles on mouse sperm chromatin using TB and showed that this metal nano-particle impedes the density of chromatin and cause abnormal chromatin condensation in mouse (6). In addition to gold nanoparticles, Rezvanfar *et al* considered the effect of selenium nanoparticles on the quality of sperm chromatin using AB and AO staining methods and showed that these nanoparticles affect the sperm DNA integrity (19). As it was mentioned before, although, the mechanisms of biological effects of gold nanoparticles need to be investigated in future, but the role of free radicals and production of mutagenic molecules following nanoparticles intake should be considered.

## Conclusion

In conclusion, our results showed that gold nanoparticles can change the mouse sperm parameters and chromatin structure and these deleterious effects are more obvious in maximum dose and chronic phase. Finally, due to the action of gold on sperm fertility potential, the use of golden jewelries and other forms of gold is not recommended especially in young men that want to have a baby.

## Note

***This article extracted from M.Sc. thesis. (Mahsa Nazari)***
